# Early introduction of complementary foods and childhood overweight in breastfed and formula-fed infants in the Netherlands: the PIAMA birth cohort study

**DOI:** 10.1007/s00394-018-1639-8

**Published:** 2018-02-22

**Authors:** Linda P. M. Pluymen, Alet H. Wijga, Ulrike Gehring, Gerard H. Koppelman, Henriëtte A. Smit, L. van Rossem

**Affiliations:** 1 Julius Center for Health Sciences and Primary Care, University Medical Center Utrecht, STR 6.131, P.O. Box 85500, 3508 GA Utrecht, The Netherlands; 2 Center for Nutrition, Prevention, and Health Services, National Institute for Public Health and the Environment, Bilthoven, The Netherlands; 3 Institute for Risk Assessment Sciences, Division of Environmental Epidemiology, Utrecht University, Utrecht, The Netherlands; 4 Department of Pediatric Pulmonology and Pediatric Allergology, Beatrix Children’s Hospital, UMCG, GRIAC Research Institute, University of Groningen, Groningen, The Netherlands

**Keywords:** Infant feeding, Solids, Overweight, Childhood

## Abstract

**Purpose:**

To investigate whether early introduction of complementary foods (CF) is associated with an increased risk of overweight during childhood, and whether this association differs between formula-fed and breastfed infants.

**Methods:**

We included 2611 participants that were born at term from a Dutch population-based birth cohort (*n* = 3963) designed to investigate the development of asthma and allergies. Parents kept records of their infant’s age when CF were first introduced. Weight and height were parent reported yearly from age 1 to 8 years, and at ages 11, 14 and 17 years. We used multivariate generalized estimating equations analysis to investigate the association between timing of CF introduction (before 4 months vs at or after 4 months of age) and overweight at ages 1–17 years.

**Results:**

Children with CF introduction before 4 months had higher odds of being overweight during childhood than children with CF introduction at or after 4 months (OR 1.32, 95% CI 1.19, 1.47). This association was observed in formula-fed infants (OR 1.51, 95% CI 1.17, 1.94) and breastfed infants (OR 1.32, 95% CI 1.19, 1.47). The duration of breastfeeding modified the association between CF introduction and overweight: children breastfed for shorter than 4 months, but not children breastfed for 4 months or longer with CF introduction before 4 months had higher odds of being overweight (OR 1.37, 95% CI 1.19, 1.57 and 1.07, 95% CI 0.87, 1.32, respectively), compared to those with CF introduction at or after 4 months.

**Conclusions:**

In children born at term, formula-fed infants and infants who were breastfed for shorter than 4 months, but not infants who were breastfed for 4 months or longer, had a higher risk of being overweight during childhood when being introduced to CF before 4 months of age.

**Electronic supplementary material:**

The online version of this article (10.1007/s00394-018-1639-8) contains supplementary material, which is available to authorized users.

## Introduction

European (including Dutch) guidelines recommend postponing the introduction of complementary foods (CF) until 4–6 months of age, while continuing breastfeeding [1, 2]. Still, circa 8–15% of infants in Europe are introduced to CF before 4 months of age [3–5].

A systematic review of prospective studies observed a higher risk of overweight and obesity at 2–12 years in infants introduced to CF before 4 months of age compared to at 4–6 months of age [6]. There are some indications that the association between early introduction of CF and overweight differs between breastfed and formula-fed infants, but results are conflicting. Moss et al. observed a higher risk of overweight at 2 and 4 years when introduced to CF before 4 months in both formula-fed infants and breastfed infants [7]. Huh et al. observed higher odds of obesity at 3 years when introduced to CF before 4 months in infants who were formula-fed or breastfed for shorter than 4 months but not in infants who were breastfed for longer than 4 months [8], while Sun et al. observed higher odds of overweight at 9–15 months when introduced to CF before 5 months, irrespective of the duration of breastfeeding [9].

Furthermore, it is unknown whether the association of early introduction of CF with overweight persists into late childhood. Since overweight in childhood may track into adulthood [10], it is important to shed light on this.

In a Dutch population-based birth cohort study, we investigated whether infants who were introduced to CF before the age of 4 months had a higher risk of being overweight during childhood than infants who were introduced to CF at or after 4 months, and whether the type of infant milk feeding and the duration of breastfeeding modified this potential association. We hypothesized that the association of introduction of CF before 4 months with overweight was different between children who were formula-fed or breastfed in infancy.

## Subjects and methods

### Study design

We used data from the Prevention and Incidence of Asthma and Mite Allergy (PIAMA) study, a population-based birth cohort study. Pregnant women were recruited from the general population in the Netherlands during their first visit to a prenatal health clinic. The baseline study population consisted of 3963 infants born in 1996–1997. The PIAMA study originally aimed to investigate the role of lifestyle and environmental factors in the development of asthma and allergies. Weight and height data had been collected annually since birth. Thus, a substantial number of overweight-related study questions could be addressed in addition to the questions that the study was originally designed to answer [11]. Details of the study are also described elsewhere [12]. Questionnaires were completed by parents during pregnancy, when the infant was 3 months old, yearly from 1 until 8 years, and at ages 11, 14 and 17 years. The study protocol was approved by the medical ethical committees of the participating institutes, and all of the parents gave written informed consent.

### Study population

For the current study, we included all children with complete data on the timing of introduction of CF (*N* = 2 752, 69.5%) and the duration of breastfeeding (*N* = 2 745). Of the 2745 children with complete data, we excluded 134 children (4.9%) who were born before 37 weeks of gestation, as these children follow a different growth pattern than those who are born at term [13] and this may influence the association under study. Thus, our sample size for the analysis consisted of 2611 children.

### Exposure: timing of introduction of complementary foods

At the infants’ age of 3 months, parents received a form on which they kept records of their infant’s age in weeks when each of the following energy-providing complementary foods (CF) were introduced for the first time: fruit, fruit juice, vegetables, milk products, wheat products, meat, fish, eggs and peanut butter. We included fruit juices as complementary foods since these can be similar to solid fruits in terms of energy content. Many children in our study received fruit juice as their first complementary food, or were introduced to fruit juice and fruit in the same week. The form was returned to the study center around the infant’s first birthday. We defined the timing of introduction of CF as the infant’s age at the earliest introduction of any CF, divided into two age categories: before 4 months (≤ 16 weeks) and at or after 4 months (> 16 weeks). This cut-off value corresponds with current guidelines which recommend to introduce CF after the age of 4 months [1, 2].

### Breastfeeding status

Data on breastfeeding during the first 3 months were collected through a questionnaire at 3 months of age. Data on breastfeeding from 4 months until 1 year of age were collected through a questionnaire at 1 year of age, as well as through a dietary assessment form prospectively filled out by the parents between the child’s age of 3 months and 1 year. We divided children into two groups: “formula-fed”, defined as infants who were never breastfed and received formula instead; and “ever breastfed”, defined as infants who received any breastfeeding. Throughout the rest of this paper, we will use the term ‘breastfed’ to refer to children who were ever breastfed. Breastfed infants were further divided into two groups: “breastfeeding for shorter than 4 months” and “breastfeeding for 4 months or longer”. The number of children introduced to CF before 4 months and who received exclusive breastfeeding for 6 months, as recommended by the WHO, was too small (*n* = 58) to analyse the association of early introduction of CF with overweight using this 6-month cut-off point.

### Outcome measure: overweight throughout childhood

In the questionnaires administered at ages 1 until 8, 11, 14 and 17 years, parents were asked to copy weight and height (which at 1 year of age is often referred to as ‘length’) data and the date of measurement from their child’s medical record, or to measure their child’s weight and height. We calculated weight-for-length and body mass index [BMI: weight (kg)/height (m)^2^] and standardized these measures into sex-specific weight-for-length percentiles and sex- and age-specific BMI percentiles, according to the reference growth curves of the Dutch Fourth Nationwide Growth Study [14]. At 1 year of age, overweight (including obesity) was defined as a weight-for-length percentile above the 90th percentile. At 2 until 17 years of age overweight (including obesity) was defined as a BMI percentile above the 90th percentile. There were too few obese children (*n* = 33/973 at 4 years, *n* = 46/1638 at 8 years, *n* = 15/1148 at 8 years and *n* = 13/634 at 16 years) to analyse them as a separate group.

### Confounders

We included the following potential confounders: sex, birth weight, maternal age at birth, maternal pre-pregnancy BMI (calculated from maternal pre-pregnancy weight in kg and height in m), maternal smoking during pregnancy (any smoking by the mother during pregnancy ≥ 4 weeks after onset of pregnancy) and maternal educational level (low: no education, primary school, lower secondary education; intermediate: intermediate vocational education, higher secondary education, pre-university education; high: higher professional education and university). We also included potential confounders based on differences observed between infants with introduction of CF before 4 months and at or after 4 months in our data: presence of siblings at birth (yes or no), method of delivery (caesarean section or vaginal delivery) and maternal weight gain during pregnancy. Maternal weight gain during pregnancy was dichotomized into excessive and no excessive weight gain using the upper cut-off of the Institute of Medicine (IOM) guidelines [15]. All covariates were retrieved from questionnaires administered during pregnancy and at the child’s age of 3 months and 1 year.

### Effect modifiers

Since there are some indications that the association between introduction of CF before 4 months and overweight may depend on the duration of breastfeeding, we included breastfeeding duration (shorter than 4 months and 4 months or longer) as a potential effect modifier. We also included maternal educational level (low, intermediate and high) as a potential effect modifier to assess whether results could be extrapolated to populations with a higher proportion of mothers with a low educational level.

### Statistical analysis

The overall association between introduction of CF before 4 months and overweight during childhood (age 1–17 years) was analysed with generalized estimating equation (GEE) models and is presented as odds ratios and 95% confidence intervals. The GEE method accounts for the correlation between repeated measurements of overweight over time within subjects and deals with missing values. The GEE model estimates the average odds ratio for the association between introduction of CF before 4 months and overweight across all ages in the entire study population. In this analysis, timing of introduction of CF was included as independent variable, with introduction of CF at or after 4 months as the reference category. In a multivariable model, we adjusted for all potential confounders described earlier.

We hypothesized that the association of introduction of CF before 4 months with overweight was different between children who were formula-fed or breastfed in infancy. We investigated this by adding an interaction term between timing of introduction of CF and breastfeeding status to the fully adjusted model and estimated the OR of the association within strata of breastfeeding. To investigate effect modification by duration of breastfeeding and maternal educational level (low, intermediate and high), we added interaction terms between timing of introduction of CF and these potential effect modifiers in separate models. In case the interactions were statistically significant at *P* < 0.20, we reported the associations in the strata separately.

In our study, we used the term complementary foods and not solid foods, since we also included fruit juices. In sensitivity analyses, we excluded fruit juices as complementary food and performed all the previous analyses using this new definition of timing of introduction of complementary foods.

Finally, we examined whether the association persisted throughout childhood. We divided children into three age groups: “preschool” (1 until 4 years). “mid-childhood” (5 until 8 years) and “late childhood” (11 until 17 years) and investigated the associations in these three ages at outcome separately. Data analysis was conducted with SAS software version 9.2 (SAS Institute, Inc, Cary, NC).

## Results

### Participants versus non-participants

Compared with the 1 352 children who were excluded from the analyses, children who were included in the analysis had a higher birth weight (3 569 vs. 3 386 g), more often had highly educated mothers (36.3 vs. 32.0%), more often received breast milk for 4 months or longer (42.4 vs. 11.7%), and less often had mothers who smoked during pregnancy (16.0 vs. 21.5%). Children included in the analysis did not differ from excluded children with regard to sex (48.5 vs. 47.5% girls), maternal overweight before pregnancy (19.5 vs. 20.4%) and maternal excessive weight gain during pregnancy (30.8 vs. 31.2%).

### Baseline characteristics of the study population

Table 1 shows the baseline characteristics of the study population (*N* = 2 611) by timing of introduction of CF and by breastfeeding status. 52.8% of formula-fed infants, 39.0% of breastfed infants, 50.5% of infants who were breastfed for shorter than 4 months and 27.8% of infants who were breastfed for 4 months or longer were introduced to CF < before 4 months. Among infants breastfed for shorter than 4 months, 81.6% of those introduced to CF before 4 months were no longer breastfed when being introduced to CF. Almost all children received fruit as first CF, either in combination with or followed by fruit juice and vegetables. In breastfed and formula-fed infants, those with introduction of CF before 4 months less often had highly educated mothers, more often had mothers with overweight before pregnancy and who smoked during pregnancy, and less often had older siblings, than those with later CF introduction.

**Table 1:** Baseline characteristics of children introduced to CF before 4 months and at or after 4 months, stratified by the type of milk feeding

Variables^a^	Formula feeding (*n* = 415)		Ever breastfeeding (*n* = 2 196)		Breastfeeding < 4 months (*n* = 1 089)		Breastfeeding ≥ 4 months (*n* = 1 107)	
	Intro CF in months		Intro CF in months		Intro CF in months		Intro CF in months	
	< 4 (*n* = 219)	≥ 4 (*n* = 196)	< 4 (*n* = 857)	≥ 4 (*n* = 1 339)	< 4 (*n* = 549)	≥ 4 (*n* = 540)	< 4 (*n* = 308)	≥ 4 (*n* = 799)
Intro CF in wks, median (IQR)	15 (12–16)	20 (18–22)	15 (12–16)	20 (18–24)	14 (12–16)	20 (18–22)	15 (13–16)	21 (18–24)
Stopped breastfeeding, age in wks, median (IQR)	0 (0)	0 (0)	12 (6–24)	22 (10–26)	7 (4–12)	8 (4–12)	28 (23–36)	34 (25–43)
Received no breastfeeding when introduced to CF	100 (219)	100 (196)	67.2 (448)	76.1 (624)	81.6 (448)	100 (540)	0.0 (0)	10.5 (84)
First CF: fruit, fruit juice and/ or vegetables	99.1 (217)	99.0 (194)	99.9 (856)	99.4 (1 331)	98.9 (543)	98.3 (531)	98.4 (303)	98.1 (784)
Maternal factors								
Age at birth in years, mean (SD)	29.7 (3.6)	30.5 (3.8)	30.1 (3.9)	31.1 (3.6)	29.8 (4.0)	30.7 (3.6)	30.6 (3.9)	31.4 (3.6)
Educational level, high	10.6 (23)	20.0 (39)	31.3 (266)	46.1 (613)	26.6 (144)	35.3 (189)	40.0 (122)	53.3 (424)
Overweight before pregnancy	32.5 (63)	25.6 (45)	20.6 (161)	16.0 (202)	22.0 (110)	18.9 (95)	18.2 (51)	14.1 (107)
Smoking during pregnancy	24.2 (53)	21.4 (41)	17.0 (144)	13.2 (176)	19.5 (106)	18.6 (100)	12.6 (38)	9.6 (76)
Excessive weight gain during pregnancy	32.3 (62)	34.1 (59)	35.5 (273)	27.3 (338)	35.3 (174)	31.1 (154)	35.6 (99)	24.7 (184)
Child factors								
Girls	46.3 (101)	52.6 (103)	48.1 (412)	48.5 (650)	47.8 (262)	45.0 (243)	48.7 (150)	50.9 (407)
Gestational age in wks, mean (SD)	39.9 (1.3)	39.8 (1.2)	40.1 (1.2)	40.1 (1.2)	40.1 (1.2)	40.1 (1.2)	40.2 (1.2)	40.2 (1.1)
Birth weight in g, mean (SD)	3534 (529)	3548 (505)	3570 (485)	3577 (460)	3562 (494)	3554 (472)	3582 (468)	3593 (452)
Birth weight > 4000 g	17.8 (39)	16.3 (32)	18.1 (155)	16.9 (226)	18.7 (103)	16.3 (88)	16.9 (52)	17.3 (138)
Birth weight < 2500 g	2.7 (6)	3.1 (6)	2.0 (17)	1.3 (17)	1.6 (9)	1.8 (10)	2.6 (8)	0.9 (7)
Delivered by Caesarean section	7.8 (17)	10.3 (20)	8.9 (75)	6.5 (86)	8.5 (46)	7.7 (41)	9.5 (29)	5.7 (45)
Siblings present at birth	57.7 (126)	61.7 (121)	43.3 (371)	55.1 (738)	41.3 (226)	51.5 (278)	47.1 (145)	57.6 (460)

*CF* complementary feeding

^a^Values are expressed as % (*n*) unless stated otherwise; missing values were 20 for educational level, 19 for smoking, 201 for maternal overweight, 237 for weight gain during pregnancy, 30 for Caesarean section, 15 for birth weight, 9 for gestational age, and 7 for maternal age

### Timing of introduction of complementary foods and overweight throughout childhood

**Fig. 1:**
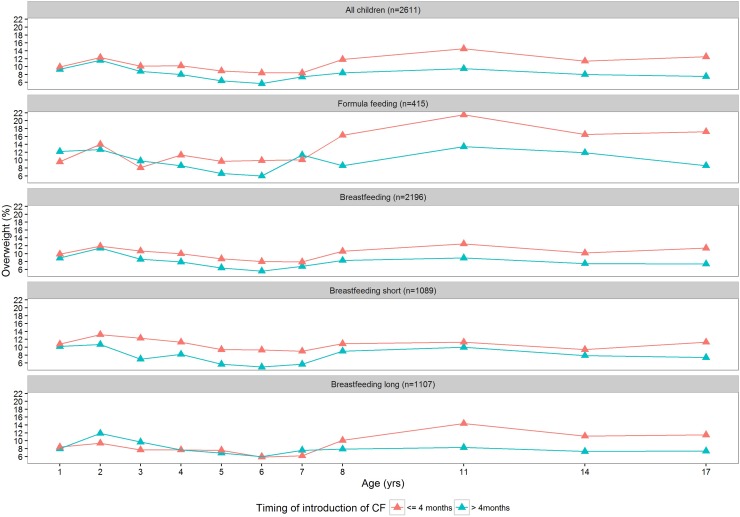
Prevalence of overweight during childhood for introduction of complementary foods before 4 months versus at or after 4 months, stratified by the type of milk feeding

The prevalence of overweight throughout childhood was higher in children with introduction of CF before 4 months than in children with CF introduction at or after 4 months Fig. 1 and Table 2, adjusted OR 1.32 (95% CI 1.19, 1.47).

**Table 2:** Odds ratio with 95% confidence interval for being overweight during childhood for introduction of complementary foods before 4 months versus at or after 4 months, in all children and stratified by the type of milk feeding

	All children (*n* = 2 611)		Formula feeding (*n* = 415)		Ever breastfeeding (*n* = 2 196)		Breastfeeding < 4 months (*n* = 1 089)		Breastfeeding ≥ 4 months (*n* = 1 107)	
	OR	95% CI	OR	95% CI	OR	95% CI	OR	95% CI	OR	95% CI
Intro CF ≥ 4 mo	1.00 (ref)		1.00 (ref)		1.00 (ref)		1.00 (ref)		1.00 (ref)	
Crude										
Intro CF < 4 mo	1.31*	1.19, 1.44	1.30*	1.04, 1.61	1.31*	1.19, 1.44	1.40*	1.21, 1.63	1.11	0.94, 1.30
Adjusted^a^										
Intro CF < 4 mo	1.32*	1.19, 1.47	1.51*	1.17, 1.94	1.32*	1.19, 1.47	1.48*	1.26, 1.73	1.02	0.86, 1.22

*CF* complementary foods

*Significant: *p* value < 0.05

^a^Model adjusted for sex, birth weight, maternal age, maternal educational level, maternal smoking during pregnancy, maternal pre-pregnancy BMI, presence of siblings, method of delivery and excessive weight gain during pregnancy

**Fig. 2:**
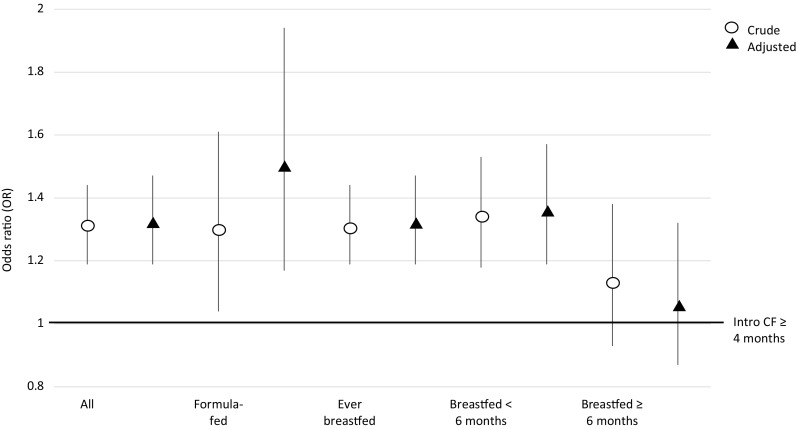
Odds ratio with 95% confidence interval for being overweight during childhood for introduction of complementary foods before 4 months versus at or after 4 months, stratified by the type of milk feeding

In both formula-fed and breastfed infants, those with introduction of CF before < 4 months were more often overweight throughout childhood than those with later introduction (adjusted OR 1.51 (95% CI 1.17, 1.94) and 1.32 (95% CI 1.19, 1.47), respectively, Table 2 and Fig. 2). The duration of breastfeeding modified the association between CF introduction and overweight: in infants who were breastfed for shorter than 4 months those with introduction of CF before 4 months had higher odds of being overweight during childhood (OR 1.48, 95% CI 1.26, 1.73) than to those with later introduction of CF; in infants breastfed for 4 months or longer CF introduction before 4 months was not associated with increased odds of being overweight during childhood (OR 1.02, 95% CI 0.86, 1.22).

Maternal educational level did not modify the association: the adjusted OR for being overweight when introduced to CF before < 4 months vs at or after 4 months in children from low-educated mothers (OR 1.53, 95% CI 1.22, 1.91) was similar to the OR in children from highly educated mothers (OR 1.51, 95% CI 1.27, 1.81, data not shown).

Results were similar when we excluded fruit juices as complementary food in sensitivity analyses (see supplemental table 1).

Finally, the association of introduction of CF before 4 vs at or after 4 months with higher odds of overweight was observed at each age period, with a statistically stronger (*P* value for interaction = 0.05) association observed in late childhood (OR 1.76, 95% CI 1.45, 2.13) than at preschool age (OR 1.28, 95% CI 1.10, 1.48) and in mid-childhood (OR 1.14, 95% CI 0.98, 1.33, data not shown).

## Discussion

### Main findings

We observed that children born at term with introduction of CF before 4 months had higher odds of being overweight between 1 and 17 years of age than children born at term with introduction of CF at or after 4 months. The duration of breastfeeding modified the association: formula-fed infants and infants breastfed for shorter than 4 months, but not infants breastfed for longer than 4 months, had higher odds of being overweight during childhood when they were introduced to CF before 4 months compared to introduction of CF at or after 4 months.

### Methodological considerations

A strength of this study is the large sample size, which enabled us to investigate the associations separately in formula-fed and breastfed infants and in infants breastfed for shorter and longer than 4 months. Another strength is the longitudinal approach which led to interesting results. The OR for the association between introduction of CF before 4 months and being overweight in late childhood was significantly higher than the OR at preschool age. Possibly, the association in late childhood is (partly) confounded by factors for which we did not adjust. For example, mothers of children who were introduced to CF before 4 months were on average lower educated and were more often overweight than mothers of children who were introduced to CF after 4 months. It is possible that children of these overweight mothers also develop a less healthy lifestyle as they become older, which increases their risk of being overweight. This may at least partially explain the stronger observed associations in late childhood. Some limitations also need to be addressed. First, a substantial number of children could not be included in our study due to missing values for the timing of introduction of CF. For the following reason, selection bias has unlikely occurred: children in our study more often had mothers with a high educational level than children who were excluded from our study, but stratified analysis showed that the association between introduction of CF before 4 months and odds of being overweight was not different between children from mothers with a low and high educational level.

Second, parents were asked to measure their child’s weight and height themselves if no weight and height data were available from medical records. We reported on the validity of parental-reported versus measured height and weight at ages 4 and 8: minor differences were observed between the child’s weight and height depending on the person who carried out the measurement [16, 17]. Since parents of children with a high BMI tended to underreport their children’s body weight, some overweight children may have been classified as non-overweight. However, as parents were unaware of the research question, we consider it unlikely that misclassified children were all introduced to CF before or after 4 months. Thus, misclassification of the outcome is most likely unrelated to the determinant, and spurious associations are, therefore, unlikely.

Third, as with all observational studies, we cannot rule out unmeasured confounding. For example, we did not know the reasons underlying the introduction of CF before 4 months. Brown et al. found that the reasons for introducing CF were associated with factors such as maternal age, educational level, parity, infant weight and sex [18], which are all factors that we adjusted for in our analyses. Therefore, we believe that unmeasured confounding could not completely explain the associations we have found.

### Findings as compared to findings from previous studies

Findings from previous studies that investigated the association between the timing of CF introduction and risk of overweight during childhood are not univocal; although effect estimates often pointed towards a higher risk of overweight with earlier introduction of CF, some studies did not observe a statistically significant association [19, 20]. Small sample sizes, differences in ages at which an association was investigated, and differences in the definition of early CF introduction may explain some of the inconsistencies between studies. For instance, an increased risk of overweight was observed in studies that used 4 months as cut-off age to define early introduction of CF [6, 21, 22].

Only few studies have investigated the association between timing of CF introduction and overweight in formula-fed and breastfed children separately. Moss and Yeaton observed an increased risk of overweight at 2 and 4 years of age with introduction of CF before 4 months in formula-fed children as well as in breastfed children [9]. We also observed this, but found that the duration of breastfeeding modified the association, with no higher risk of overweight observed in children breastfed for 4 months or longer. This is in line with findings reported by Huh et al. [10], but not with findings reported by Sun et al. where introduction of CF before 4 months was associated with higher odds of overweight at 9–15 months in infants breastfed for shorter than 4 months as well as in infants breastfed for 4 months or longer [11]. However, Huh et al. combined children breastfed for shorter than 4 months with formula-fed children, and were thus unable to report whether the association was different between these two groups. The fact that we observed differences in association between formula-fed infants, infants breastfed for shorter than 4 months and infants breastfed for 4 months or longer may partly explain the discrepancy in previous findings, especially if the proportion of formula-fed and breastfed children differed between studies.

### Possible explanations for findings

As mentioned earlier, the reasons for introducing CF earlier than recommended may partly explain the observed association in formula-fed children and in children breastfed for shorter than 4 months if these reasons are related to the odds of being overweight during childhood as well.

Alternatively, it has been suggested that formula-fed infants are less able to self-regulate their energy intake than breastfed infants [23]. Evidence with respect to the timing of introduction of CF is scarce: two observational studies found that early introduction of CF displaced milk intake among breastfed infants but not among formula-fed infants [24, 25]. However, estimating intake of breast milk is challenging. Besides, a randomized controlled trial found that early introduction of CF at 3–4 months (vs. 6 months) was associated with greater displacement of formula milk, suggesting that formula-fed infants are able to self-regulate energy intake [26].

### Relevance of findings and research perspectives

We observed that the association between introduction of CF before 4 months and overweight persisted up to the age of 17 years. This is worrisome, as overweight can track into adulthood [12], where it may lead to hypertension, diabetes mellitus type II and increased risk of cardiovascular diseases.

Since we only included children born after 37 completed weeks of gestation, our findings may not be generalizable to children who were born preterm. Although the children in this study were born 20 years ago, our results are still relevant for children born nowadays. The prevalence of overweight in our study was comparable to the prevalence of overweight in the general Dutch population, and has been stable throughout the last 20 years (11.5% of boys and 12.1% of girls aged 4–20 years were overweight in 1997 versus 12.7% of boys and 11.5% of girls in 2015) [27]. In 1997, Dutch guidelines recommended to introduce CF at 6 months of age, but earlier introduction of CF from 4 months of age was allowed [28]. This recommendation is similar to the current recommendation of introducing CF between 4 and 6 months of age [1, 2]. We observed that more than 40% of children in the PIAMA study were introduced to CF before 4 months of age. In other Dutch birth cohort studies with year of birth between 2001 and 2005, only 5–11% of infants were introduced to CF before 4 months of age [4, 5, 31]. Possibly, adherence to the guidelines has improved throughout the years, as is seen for breastfeeding; the percentage of Dutch mothers who initiate breastfeeding has increased from 70% in 1996 [29] to 80% in 2015 [30], and the percentage of mothers who exclusively breastfeed for at least 6 months has increased from circa 6% in 1996 [29] to 39% in 2015 [30]. Unfortunately, the Dutch National Food Consumption Survey does not assess food intake of children younger than 1 year of age, and thus there are no current data available describing the prevalence of early introduction of CF in The Netherlands. Still, a prevalence of early introduction of CF of 5–11% is worrisome, since this equals circa 8600−19,000 Dutch infants each year being introduced to CF before 4 months of age who may have an increased risk of developing overweight during childhood.

It should be mentioned that the guidelines on the optimal timing of introduction of CF are mainly based on evidence regarding the risk of developing food allergies [32], and more recent studies have suggested that earlier introduction of allergenic CF, rather than delayed introduction as was thought, may be protective against food allergies [33–37]. In general, children are first introduced to non-allergenic CF such as fruits and vegetables. We cannot think of a reason why the association between introduction of CF before 4 months and the odds of being overweight would be different for allergenic and non-allergenic CF. If guidelines are updated based on this recent evidence, and more children are introduced to CF before 4 months, then our study suggests that this may increase the risk of overweight in children who receive formula feeding and in children who receive breast milk for shorter than 4 months.

## Conclusion

Among children born at term, formula-fed children and children breastfed for shorter than 4 months, but not children who were breastfed for 4 months or longer, had a higher risk of being overweight during childhood when being introduced to CF before 4 months as compared to later introduction of CF. In light of the current debate about the optimal timing of introduction of CF with regard to allergy prevention, our results need to be considered if guidelines on infant feeding practices are updated.

## Electronic supplementary material

Below is the link to the electronic supplementary material.

Supplementary material 1 (DOCX 17 KB)
