# Supplementary material for: Early introduction of complementary foods and childhood overweight in breastfed and formula-fed infants in the Netherlands: the PIAMA birth cohort study
Source: Eur J Nutr. 2018 Feb 22;57(5):1985–93. doi: 10.1007/s00394-018-1639-8 (PMC6060808; doi:10.1007/s00394-018-1639-8)
Supplement: Supplementary file 1 — Supplementary material 1 (DOCX 17 KB) [file 394_2018_1639_MOESM1_ESM.docx]

|  | All children  (n=2 611) | | Formula feeding  (n=415) | | Ever breastfeeding  (n=2 196) | |  | |  | |
| --- | --- | --- | --- | --- | --- | --- | --- | --- | --- | --- |
|  |  | |  | |  | | Breastfeeding < 4 months  (n=1 089) | | Breastfeeding ≥ 4 months  (n=1 107) | |
|  | OR | 95% CI | OR | 95% CI | OR | 95% CI | OR | 95% CI | OR | 95% CI |
| Intro CF ≥ 4 mo. | 1.00 (ref) | | 1.00 (ref) | | 1.00 (ref) | | 1.00 (ref) | | 1.00 (ref) | |
| Crude: |  | |  | |  | |  | |  | |
| Intro CF < 4 mo. | 1.21* | 1.10, 1.33 | 1.30* | 1.04, 1.61 | 1.16* | 1.04, 1.29 | 1.34* | 1.16, 1.55 | 0.94 | 0.79, 1.10 |
| Adjusted^1^: |  | |  |  |  | |  |  |  |  |
| Intro CF < 4 mo. | 1.22* | 1.10, 1.35 | 1.51* | 1.17, 1.94 | 1.17* | 1.04, 1.31 | 1.39* | 1.19, 1.63 | 0.91 | 0.77, 1.09 |

CF, complementary foods

^1^ Model adjusted for sex, birth weight, maternal age, maternal educational level, maternal smoking during pregnancy, maternal pre- pregnancy BMI, presence of siblings, method of delivery and excessive weight gain during pregnancy

* Significant: p-value <0.05
